# Novel tools integrating metabolic and gene function to study the impact of the environment on coral symbiosis

**DOI:** 10.3389/fmicb.2014.00448

**Published:** 2014-08-21

**Authors:** Mathieu Pernice, Oren Levy

**Affiliations:** ^1^Plant Functional Biology and Climate Change Cluster, University of Technology, SydneySydney, NSW, Australia; ^2^The Mina and Everard Goodman Faculty of Life Sciences, Bar-Ilan UniversityRamat Gan, Israel

**Keywords:** coral, symbiosis, *Symbiodinium*, genomics, stable isotopes

## Abstract

The symbiotic dinoflagellates (genus *Symbiodinium*) inhabiting coral endodermal tissues are well known for their role as keystone symbiotic partners, providing corals with enormous amounts of energy acquired via photosynthesis and the absorption of dissolved nutrients. In the past few decades, corals reefs worldwide have been increasingly affected by coral bleaching (i.e., the breakdown of the symbiosis between corals and their dinoflagellate symbionts), which carries important socio-economic implications. Consequently, the number of studies focusing on the molecular and cellular processes underlying this biological phenomenon has grown rapidly, and symbiosis is now widely recognized as a major topic in coral biology. However, obtaining a clear image of the interplay between the environment and this mutualistic symbiosis remains challenging. Here, we review the potential of recent technological advances in molecular biology and approaches using stable isotopes to fill critical knowledge gaps regarding coral symbiotic function. Finally, we emphasize that the largest opportunity to achieve the full potential in this field arises from the integration of these technological advances.

## INTRODUCTION

Reefs based on scleractinian corals are among the most productive and biologically diverse marine ecosystems on Earth (Moberg and Folke, 1999). At the heart of the success of corals is their symbiosis with dinoflagellate algae (zooxanthellae), which live within their tissues and provide each coral polyp with a wider metabolic repertoire (Anthony and Hoegh-Guldberg, 2003; Houlbreque and Ferrier-Pages, 2009). This fundamental symbiosis is known to enhance the ability of corals to synthesize a calcium carbonate skeleton (Gattuso et al., 1999), the structural basis of coral reef ecosystems, in an environment where nutrients are mostly limited.

Since 1979, populations of scleractinian corals have been reported as increasingly affected by mass coral bleaching, which involves the breakdown of the symbiosis between the cnidarian host and the dinoflagellate symbionts (Hoegh-Guldberg, 1999). Given that the economic value of coral reefs has been estimated around US $375 billion per year (Costanza et al., 1997) and that coral reefs support over 500 million people through the services and food that they provide, losing corals from reef systems would have substantial impacts on coastal populations worldwide. Consequently, many studies have focused on the causes and mechanisms of the disruption of this symbiosis in the past decades (more than 18,000 articles are available on Google scholar since 1990). However, despite the undoubted causal link between environmental stresses and bleaching, capturing the complexity of the interaction between coral-dinoflagellate symbiosis and its surrounding environment remains challenging, especially given that (i) the environment is complex and defined by a multitude of factors and (ii) the study of coral symbiosis is complex and hampered by the intertwined nature of coral-dinoflagellate symbiosis. Considering the complexity of coral symbiosis, here we identify specific knowledge gaps within the functioning of coral symbiosis, including immune defenses and metabolism, and argue that recent technological advances provide better tools to understand how the environment affects these functional processes.

## CORALS GROW IN A NARROW BAND OF ENVIRONMENTAL CONDITIONS, NEAR THEIR PHYSIOLOGICAL LIMITS

Corals thrive in tropical waters, which happen to be warm, clear and generally oligotrophic (Muscatine and Porter, 1977). This narrow and consistent band of environmental conditions signifies that corals live and grow best near their physiological limits, especially with regards to three main factors: temperature, light and nutrients. Among these factors, the interaction between temperature and light has been intensively studied over the last decades because of a major interest in understanding thermal and light stress-related bleaching phenomena (Hoegh-Guldberg, 1999). One of the first sites of damage is the symbiont photosystem II apparatus (Lesser, 2006), a key component of photosynthetic pathways located within the chloroplast of *Symbiodinium*. This photosynthetic dysfunction results in the excessive production of reactive oxygen species (ROS) in the symbiont and promotes the degradation of host mitochondria, providing another potential site of harmful ROS production and oxidative stress (Dunn et al., 2012a). The excess ROS damages essential biological macromolecules and cellular structures, initiating a cascade of innate immune responses, which subsequently result in the release and/or degradation of the symbiotic dinoflagellates. Histological studies have revealed two possible mechanisms of symbiont degradation: (i) symbionts are degraded from the effects of ROS via programmed cell death (Strychar et al., 2004) or (ii) the coral host actively destroys the symbionts and ultimately expels them (Dunn et al., 2007b). The cellular mechanisms of coral bleaching have been the focus of many studies since the 1990’s, which are already well reviewed (for review, see: Weis, 2008). However, despite the clear involvement of cellular mechanisms such as exocytosis, host cell detachment, apoptosis, and necrosis, the cascade of immune responses and the modulation of cell death pathways leading to bleaching remain unsolved.

## TECHNOLOGICAL ADVANCES IN MOLECULAR BIOLOGY PROGRESS TOWARD UNRAVELING GENE FUNCTION

By allowing researchers to simultaneously investigate genes and their level of expression, genomics and transcriptomics approaches have greatly improved our understanding of coral bleaching. In the late 2000s, the first microarrays studies had a tremendous impact on revealing the cellular foundation of thermal stress-induced coral bleaching (Desalvo et al., 2008; Rodriguez-Lanetty et al., 2009; Bellantuono et al., 2012). In the first medium–scale transcriptomics coral study, Desalvo et al. (2008) used a cDNA microarray containing more than 1300 genes of the coral *Montastraea faveolata* to measure gene expression changes associated with thermal stress*.* Their results suggested that oxidative stress in thermal-stressed corals causes a disruption of Ca^2+^ homeostasis and the initiation of cell death via apoptosis and necrosis. In a following study, Rodriguez-Lanetty et al. (2009), examined the effect of thermal stress on the early transcriptional response of aposymbiotic larvae of the reef-building coral *Acropora millepora* and show that elevated temperature compromise some critical components of the coral immune defenses including a mannose-binding C-type lectin. More recently, microarrays studies demonstrated that coral host transcriptomic states are correlated with different *Symbiodinium* genotypes (Desalvo et al., 2010) and that potential “early warning genes” and “severe heat-related genes” could be detected as a result of high heat stress (Maor-Landaw et al., 2014). The findings from Maor-Landaw et al. (2014) also suggest that during short-term heat stress, *S. pistillata* may divert cellular energy into mechanisms such as the ER-unfolded protein response (UPR) and ER-associated degradation (ERAD) at the expense of growth and biomineralization processes in an effort to survive and subsequently recover from the stress. The emergence of next-generation sequencing technologies has further increased the speed of coverage and decreased the cost through massively parallel sequencing methods. As a result, large–scale transcriptomics are well established as part of the coral biologist’s “toolbox” (Moya et al., 2012) and a total of approx. 600,000 sequences with more than 86,000 unique blast matches are available from various coral species through six assembled transcriptomes and one fully assembled genome (Shinzato et al., 2011) in databases such as systems biology of symbiosis^1^, compagen^2^, or cnidarian^3^. Genomic information on *Symbiodinium* are more scarce, due primarily to its very large genome size (approx. 2–4 Gb) and repetitive DNA (Leggat et al., 2011). However, since 2007, several expression sequence tags (ESTs) and subsequent gene expression studies were generated (Leggat et al., 2007; Rosic et al., 2010, 2011a,b), and more recently, the gene structure of the dinoflagellate has been further revealed by the draft assembly of the *S. minutum* nuclear genome (Shoguchi et al., 2013). Despite these enormous progresses, how the environment and genes ultimately interact to affect immune defenses and cell death in coral symbiosis remains poorly understood. Currently, the biggest challenges in this field are (i) to organize the growing body of molecular information into a clear mechanistic framework and testable hypotheses with a direct link to function and (ii) to direct hypothesis-driven research to aid in the examinations of gene function. While the first challenge is conceptual and beyond the scope of this paper, the latter is more technological and could be achieved using advanced functional approaches to silence/knockdown gene expression. In this respect, previous studies in the freshwater cnidarian *Hydra magnipapillata* have explored the use of the reverse-genetics technique RNA interference (RNAi), which consists of introducing a synthetic double strand of RNA into cells to selectively induce gene suppression. Unfortunately, this method has had limited success; the RNA delivery via electroporation often damages tissues and cells (Lohmann et al., 1999; Smith et al., 2000; Cardenas and Salgado, 2003). In a pioneering gene silencing study for a symbiotic cnidarian, Dunn et al. (2007a) reported the use of RNAi and chemical transfection delivery to suppress the expression of a gene coding for caspase, a proteolytic enzyme triggering cell death and bleaching in the symbiotic sea anemone *Aiptasia pallida.* Although their method was effective, the decrease obtained in caspase activity was not absolute (only 30%), most likely related to the instability or poor delivery of siRNA *in vivo*. Genetic modification is still in its infancy in symbiotic cnidarians, and efforts at using siRNA for gene silencing have often been hampered by the difficulty of effectively introducing it into cells of interest. As a result, no other attempts to use siRNA in corals have been published thus far.

Nanotechnology is a relatively new discipline, which in the last years is starting to be used for addressing questions related to biological systems. Thanks to nanotechnology, new materials can be developed that have new properties compared to existing properties. Nanomaterials have been shown to possess distinctive properties that contribute to promising applications in several fields. To give an example, fluorescent semiconductor nanoparticles (NPs) suffer less from photobleaching than conventional fluorophores. Thus, single-molecule based tracking of fluorescently labeled membrane-bound proteins received a great boost by moving from connectional fluorophores to fluorescent NPs (Bouzigues et al., 2007). The small size of the NPs gives them a high surface area-to-volume ratio and facilitates the interaction with several types of chemical species. NPs are also excellent candidates for drug delivery due to their capacity to interact with biomolecules and their possibility to be loaded with specific cargo (Bouzigues et al., 2007; Conde et al., 2012). Although NPs can be useful devices for delivering specific cargo *in vitro* and *in vivo* and their uptake is easily carried out, the specific control of the cargo release is still a challenge. Some years ago, a new concept was presented in which hollow particles, typically on the micrometer scale (so called polyelectrolyte capsules), were used as a carrier system. The cavity of the capsules can be loaded with a large variety and high quantity of cargo (De Koker et al., 2009; De Cock et al., 2010; Conde et al., 2012). If the walls of the capsules are modified with Au NPs, an optothermal opening can be made, providing the possibility to carry out a controlled release of the cargo (Skirtach et al., 2006; De Koker et al., 2009). This release is similar to the concept of caged Ca^2+^, in which Ca^2+^ ions are released from a chelator upon a flash of light. Capsules, however, allow for the release of a larger variety of cargo molecules, such as small drugs, proteins, or mRNA. Capsules can range in size from hundreds of nanometers to a few micrometers, depending on the size of the template that was used in synthesis (Skirtach et al., 2006; Wang et al., 2008). When less specific control of the release is required, biodegradable capsules are also a good alternative. It has been demonstrated that biodegradable capsules are degraded inside of the lysosome of cells, where their cargo is then released*.* Thus, nanotechnology can provide sophisticated carrier systems ranging from a few nanometers to a few micrometers, which allows for the controlled release of biologically active cargo. This next generation of carrier systems could be a game changer in the understanding of bleaching mechanisms, as they also include NPs with new biomaterials developed to fit the chemistry, biophysical structure, and biological function of siRNA. Many research groups are already reporting improved stability and delivery efficiency of siRNA (for review, see: Kozielski et al., 2013). However, in model organisms such as Nematods or Zebrafish, a reliable means of RNAi-mediated gene knockdown remains elusive (Kelly and Hurlstone, 2011). As such, a simpler alternative for gene knockdown, such as morpholino antisense oligos, is still the most effective method of gene suppression in Zebrafish. Morpholinos act by binding and blocking access to target mRNA (Nasevicius and Ekker, 2000). Since their first introduction in the early 2000’s, they been used in a range of model organisms, including sea urchin, ascidian, zebrafish, frog, chick, and mouse providing a relatively simple and rapid method to study gene function (Huang et al., 2012; for review see: Heasman, 2002). Although these better ways for gene suppression still require optimization and validation in coral symbiosis, their future development could be critical to address important hypotheses about gene function, such as the existence of symbiosis-specific genes.

## IMPORTANCE OF NUTRIENTS AND METABOLIC FUNCTION IN CORAL SYMBIOSIS

Given the nutritional role of the symbionts, another intriguing question—and subsequent knowledge gap in coral symbiosis—arises as to whether bleaching may in part reflect a change in the nutritional status of the host–symbiont interaction. In this respect, recent studies have highlighted strong correlations between feeding, sustained photosynthetic activity and reduced bleaching (Ferrier-Pages et al., 2010; Beraud et al., 2013). Coral symbiosis requires the delicate balance of exchanged compounds between the symbiotic partners. The photosynthetically fixed carbon compounds translocated by the dinoflagellate symbiont to the host consist largely of non-nitrogenous compounds, such as glycerol, glucose, and succinate (Venn et al., 2008). These compounds are often referred to as “junk food,” as they directly support coral respiration and mucus production (Wild et al., 2004) but can only be used for coral growth when nitrogen and phosphorus are available from another source (Ferrier-Pages et al., 2010). Consequently, the ability to assimilate nitrogen and phosphorus by feeding on plankton (Houlbreque and Ferrier-Pages, 2009) or by absorbing nutrients dissolved in seawater, with a preference for phosphate, ammonium and nitrate (Grover et al., 2002), is a crucial attribute of the coral symbiosis.

Ecological stoichiometry is an increasingly broad field of research that evaluates how the relative quantity of specific chemical elements (C:N:P ratio) constrains or facilitates the movement of nutrients through an ecosystem (Sterner and Elser, 2002). Given that elemental ratios are unit less, using a stoichiometric approach allows for the tracking of the same response at scales where quantities differ greatly (for example, from single cells to community). Recent advances in this field of research have demonstrated an intimate link between biomass stoichiometry, environmental conditions and nutrient fluxes in a broad range of ecosystems (Taylor and Townsend, 2010). The same is certain to be the case for coral and coral reefs; the changes in biomass stoichiometry, driven by changes in macromolecular composition, reflect coral physiology (e.g., growth rate or accumulation of storage compounds) and *in situ* environmental conditions (e.g., temperature, nutrients, or light availability). Shifting the balance of supply versus demand for nitrogen and carbon, for example due to an environmental perturbation, may well result in the disruption of the coral–symbiont relationship, which is referred to as bleaching. The development of research investigating environmental impacts on the metabolic function of coral reefs is particularly necessary given that (i) reefs are rapidly deteriorating worldwide and that (ii) most coral reef research does not consider biomass stoichiometry and metabolic function when studying bleaching. Providing redress for such an omission may be critical in understanding the issue of coral reef degradation.

## TECHNIQUES BASED ON STABLE ISOTOPES TO QUANTIFY METABOLIC ACTIVITY *IN SITU*

The intertwined nature of the coral-dinoflagellate endosymbiosis has long hampered research on coral metabolic function, as studies often suffer from potential cross-contamination between coral host and dinoflagellate fractions (Yellowlees et al., 2008). In this context, the recent development of approaches combining incubations with stable isotopes and analysis of elements, DNA, RNA and other biomarkers has been a revolutionary step, allowing the detection of metabolically active microbes in their natural habitat (for a review on these techniques and their application in microbiology Musat et al., 2008; Orphan and House, 2009; Wagner, 2009; Murrell and Whiteley, 2011). Stable isotope probing (SIP) is a relatively recent method to track the metabolic fate of a compound “isotopically labeled.” In a seminal study, Radajewski et al. (2000), reported that 13C-DNA, produced during growth methylotrophic bacteria on a 13C-enriched carbon source could be resolved from 12C-DNA by density-gradient centrifugation, allowing both taxonomic and functional characterization by gene probing and sequence analysis (Radajewski et al., 2000). This method was described as DNA-SIP, and was soon after followed by RNA-SIP (Manefield et al., 2002), which provided another novel way to link the phylogeny of microorganisms to their function. Since then, SIP has been developed with the use of different stable isotopes including 15N and 18O (for review, see Murrell and Whiteley, 2011).

Stable isotope probing techniques are not the only way to track metabolic incorporation of stable isotopes. Metabolomics, which involves the quantitative analysis of all metabolites present in cells and tissues, can be used in combination with stable isotope and has the potential to play a key role in understanding coral symbiosis (for review, see: Gordon and Leggat, 2010). In a recent study, Dunn et al. (2012b) used stable isotopic incorporation from dissolved inorganic carbon (NaH^13^CO_3_) combined with HPLC-MS to investigate the lipogenesis in symbiotic cnidarian. Interestingly, their results indicated that fatty acids derived from photosynthetically fixed carbon were not used directly in host lipogenesis, suggesting that additional sources of carbon, such as host respiration and heterotrophy may be especially important for the lipogenesis of fatty acids in the cnidarian host. Another technique that over- comes the use of stable isotopes to detect metabolically active microbes in their natural environment is nano-scale secondary ion mass spectrometry (NanoSIMS). With NanoSIMS, secondary ions are extracted from the surface of a sample under the impact of a primary ions beam and subsequently analyzed using mass spectrometry providing imaging and quantification of up to seven isotopes of elements simultaneously (Wagner, 2009). When combined with stable isotopes incubation, NanoSIMS can be used to measure the relative metabolic contribution of different symbiotic partners (i.e., metabolic rates of individual host and symbiont cells) with single cell resolution (Pernice et al., 2012; Kopp et al., 2013). This technique can also be used in concert with *in situ* hybridizations to simultaneously identify individual cells and quantify their substrate uptake (Behrens et al., 2008; Musat et al., 2008). Although this combination of NanoSIMS and *in situ* hybridization has never been applied to coral, studies integrating these powerful methodologies can significantly improve our understanding of the functional diversity that exists at the very heart of reef-building corals (Pernice et al., 2014).

By allowing direct empirical evaluation of a proposed hypothesis, these functional approaches combining incubations with stable isotopes and biomarkers or elemental analysis can help addressing fundamental questions in coral symbiosis. Among others, an interesting hypothesis that remains poorly addressed so far concerning coral symbiosis, is whether photosynthetically fixed carbon may directly contribute to calcification of coral skeleton? Indeed, previous studies have demonstrated that photosynthesis and skeleton formation are tightly coupled in zooxanthellate scleractinian corals, calcification being, on average, three times higher in light than in darkness [for review, see Gattuso et al. (1999)]. However, the details of carbon supply to the calcification process are almost unknown. In this respect, elemental analyses combined with incubations using multiple stable isotopes focusing on skeletal formation [e.g., using ^86^Sr (Houlbreque et al., 2009)] and fixation of carbon via photosynthesis [e.g., using NaH^13^CO_3_ (Pernice et al., 2014)] have great potential to address this hypothesis and may be critical in understanding the link between photosynthesis and skeletal formation.

**FIGURE 1:**
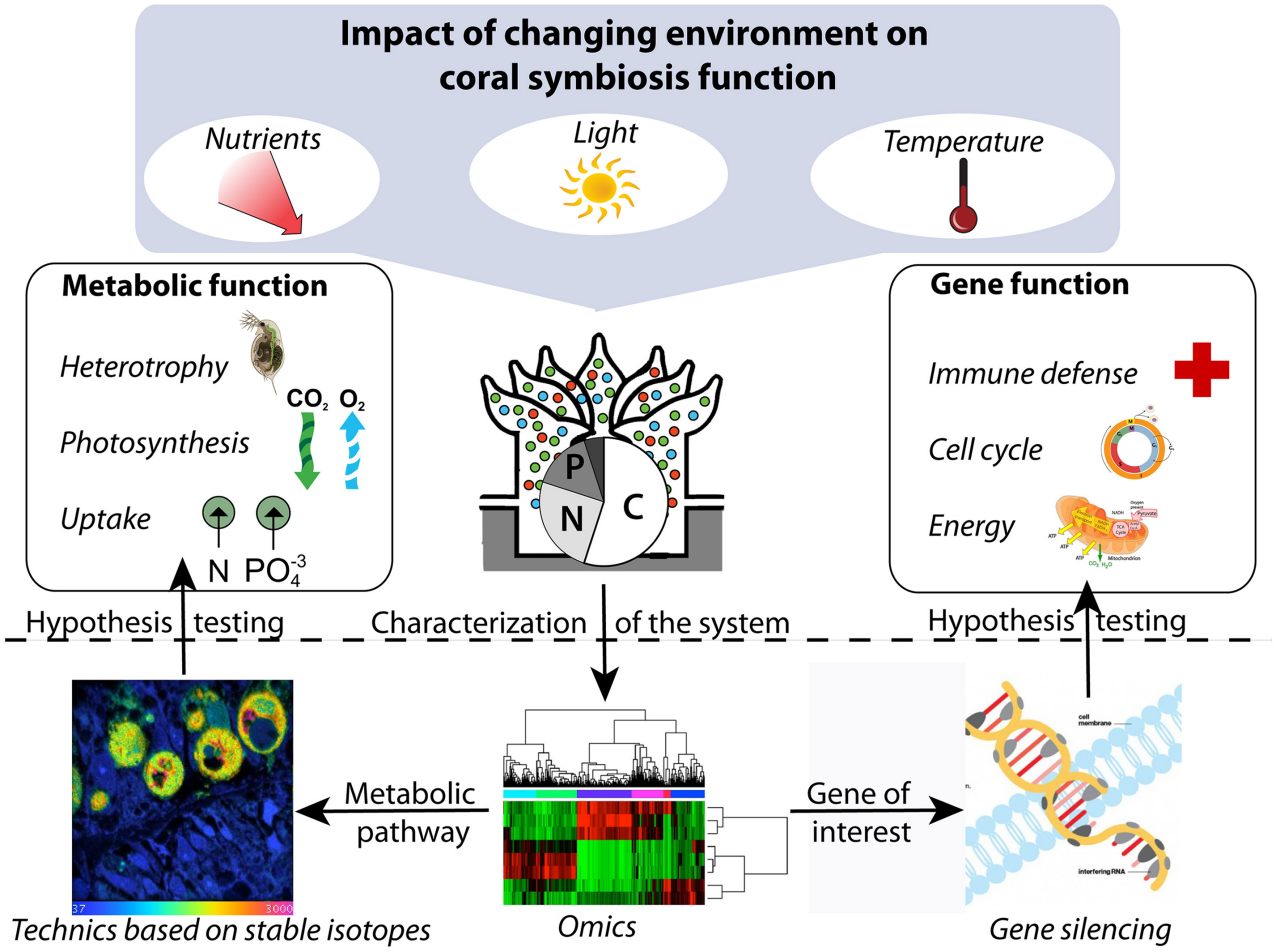
**Conceptual framework integrating metabolic and gene function to study the impact of the environment on coral symbiosis.** The “omics” approaches can be used to describe the system under different conditions and to identify genes and metabolic pathways of interest, which can subsequently be targeted by gene silencing and technics based on stable isotopes, respectively, to direct hypothesis-driven research.

When integrated with recent advances in molecular biology, including “omics” approaches and next generation siRNA delivery systems, approaches based on incubation with stable isotopes could also provide important opportunities for system biology (Raes and Bork, 2008; **Figure 1**). Discovering the relationships between gene functions and the sum of all metabolic processes (i.e., nutrients and energy cycling) occurring within symbiotic corals could be an especially important next step to better understand the biology and functioning of this symbiosis.

## Conflict of Interest Statement

The authors declare that the research was conducted in the absence of any commercial or financial relationships that could be construed as a potential conflict of interest.

## Footnotes

1 http://www.auburn.edu/~santosr/symbiosys.htm

2 http://www.compagen.org/links.html

3 http://data.centrescientifique.mc/blast/?option=home.php
